# ROS-Mediated Cardiomyocyte Proliferation and Myocardial Regeneration: Mechanisms and Targeted Strategies for Ischemic Heart Disease

**DOI:** 10.3390/jcdd13030105

**Published:** 2026-02-25

**Authors:** Mengqi Chen, Tingting Liu, Fangling Sun, Xin Tian, Wenrong Zheng, Zixin Zhu, Wen Wang

**Affiliations:** 1 Department of Experimental Animal Laboratory, Xuanwu Hospital of Capital Medical University, Changchun Street, Xicheng District, Beijing 100053, China; kikipha@mail.ccmu.edu.cn (M.C.); sun_fangling@163.com (F.S.); xintian@xwhosp.org (X.T.); zwr15710022830@163.com (W.Z.); zixin-zhu@163.com (Z.Z.); 2 Beijing Geriatric Medical Research Center, Beijing 100053, China; 3 Beijing Institute for Brain Disorders, Beijing 100069, China

**Keywords:** reactive oxygen species, heart regeneration, cardioprotective therapy, redox homeostasis, ischemic heart disease

## Abstract

Cardiovascular disease (CVD) persists as the leading cause of global mortality, with adult mammalian hearts exhibiting limited regenerative capacity. Although cardiomyocytes (CMs) can re-enter the cell cycle and undergo DNA synthesis in response to injury, they fail to complete mitosis and cytokinesis, resulting in a functional blockade of productive proliferation following ischemic or aging-related injury. Reactive oxygen species (ROS) exhibit a context-dependent duality in cardiac regeneration: while maintaining redox homeostasis and supporting developmental signaling at physiological concentrations, pathological ROS accumulation exacerbates myocardial decline by inducing DNA damage response (DDR)-mediated cell cycle arrest at G2/M phase, along with structural and functional impairments. This review examines the mechanisms of ROS generation—from its cellular origins to its molecular drivers—in ischemic heart disease, and explores the modulation of regenerative signaling by oxidative stress. We further critically assess emerging therapeutic interventions targeting ROS-mediated myocardial regeneration. By delineating the functional roles of ROS in cardiac injury and repair, this review provides a mechanistic and translational framework for developing redox-based therapies aimed at promoting cardiomyocyte proliferation and myocardial regeneration after ischemic injury.

## 1. Introduction

CVD remains the leading cause of death worldwide, posing a major public health challenge. Driven by an aging population and lifestyle changes, its rising prevalence is expected to place unprecedented economic and operational burdens on healthcare systems globally. Following ischemic myocardial injury, necrotic cardiac tissue is replaced by fibrotic scar, leading to impaired contractility and initiating pathological remodeling that often progresses to heart failure (HF). Affecting over 64 million people worldwide, HF represents the end-stage of this process and remains a critical unmet medical need [1]. Although neonatal mammals briefly retain some capacity for cardiac regeneration after birth, this potential is rapidly lost during maturation [2]. The limited regenerative ability of the adult heart hinders effective compensation for cardiomyocyte loss after myocardial infarction (MI) or myocarditis, frequently resulting in HF [3].

ROS are oxygen-derived metabolites with strong oxidative properties, including free radicals (e.g., superoxide anion [O_2_^−^·] and hydroxyl radical [HO·]) and non-radical oxidants (such as hydrogen peroxide [H_2_O_2_] and peroxynitrite [ONOO^−^]) [4,5]. In the cardiovascular system, ROS are generated from multiple sources, including NADPH oxidases (NOX2/4), uncoupled nitric oxide synthase (NOS), and mitochondrial electron transport chain complexes [6,7,8,9,10]. Mitochondria represent the major cellular source of ROS (accounting for approximately 90%), wherein electron leakage from complexes I and III of the respiratory chain—aggravated under ischemic conditions—generates superoxide, resulting in oxidative damage to mitochondrial DNA, respiratory impairment, and activation of pro-hypertrophic signaling pathways [7,8,11].

ROS play a dual role in cardiac physiology, with context-dependent effects. At physiological concentrations (e.g., H_2_O_2_ gradients of 10–100 nM), they serve as essential second messengers regulating proliferation, differentiation, and inflammatory signaling [6,7]. ROS play a dual role in cardiac physiology, with context-dependent effects. At physiological concentrations (e.g., H_2_O_2_ gradients of 10–100 nM), they serve as essential second messengers regulating proliferation, differentiation, and inflammatory signaling [7,12,13,14]. While endogenous antioxidant systems—including enzymatic mechanisms (SOD, CAT, GPx) and non-enzymatic scavengers—maintain redox balance under normal conditions [7,13], this equilibrium is disrupted during myocardial ischemia or aging, resulting in oxidative stress [7]. Transcriptional regulators such as Nrf2 play critical roles in coordinating this antioxidant defense network [14], while deficiency in Meis1 compromises ROS clearance capacity and promotes metabolic dysregulation [15,16].

Accumulating evidence indicates that ROS-mediated oxidative stress is a key mechanism driving the loss of cardiac regenerative capacity within the first week after birth [17]. Significant redox dysregulation is also observed following ischemic injury [18]. This dual role of ROS—as essential signaling molecules at physiological levels and as pathological effectors when dysregulated—forms the central paradigm for understanding their involvement in myocardial regeneration [6]

This review systematically examines the mechanisms of ROS generation in the context of ischemic heart disease, and analyses the regulation of oxidative stress in the context of regulation of cardiac regeneration. We evaluate therapeutic strategies targeting ROS metabolism and redox microenvironment optimization to enhance cardiac regeneration and cardiac function. These investigations advance understanding of regenerative capacity decline precipitously with maturation while proposing novel ROS-modulated approaches for myocardial protection and repair.

## 2. Mechanisms of ROS-Mediated Regulation in Myocardial Regeneration

**Figure 1:**
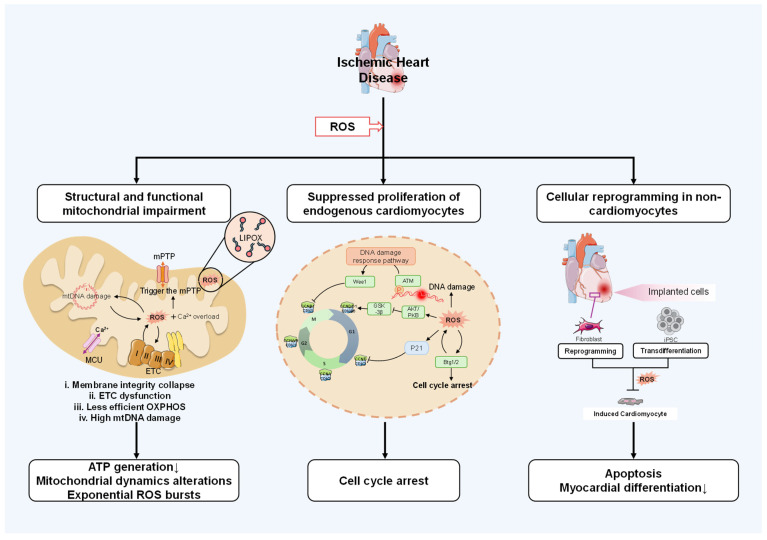
The pathological mechanisms of ischemic heart disease driven by ROS.

Building upon the established dual role of ROS as signaling mediators and pathological effectors in myocardial regeneration, we now examine the specific molecular and cellular mechanisms through which ROS orchestrate this finely balanced process. The spatiotemporal precision of redox signaling is a critical determinant of cardiac cell fate; its dysregulation initiates detrimental cascades, whereas controlled signaling supports regenerative pathways. This section focuses on three core mechanisms: ROS-induced mitochondrial dysfunction and its consequences for cellular bioenergetics and redox homeostasis; oxidative suppression of endogenous cardiomyocyte proliferation via DNA damage and resulting proliferative arrest; and ROS-mediated reprogramming of non-cardiomyocytes toward cardiac phenotypes (Figure 1). Clarifying these regulatory networks is indispensable for designing targeted therapies that preserve beneficial ROS signaling while curtailing harmful effects, thereby promoting innovative strategies for heart regeneration.

Reactive oxygen species (ROS) generated after myocardial infarction drive three major pathological pathways: (1) Mitochondrial Dysfunction: ROS induce opening of the mitochondrial permeability transition pore (mPTP), exacerbated by Ca^2+^ overload (via MCU) and lipoxygenase (LIPOX). This leads to membrane disruption, electron transport chain (ETC; Complexes I–V) impairment, reduced oxidative phosphorylation (OXPHOS), mtDNA damage, and aberrant mitochondrial dynamics, collectively diminishing ATP production and amplifying ROS generation. (2) Inhibition of Cardiomyocyte Proliferation: ROS cause DNA damage, activating the DNA damage response (involving ATM, Wee1) and elevating p53 and P21, which induce proliferative arrest through the p16/Rb pathway, thereby blocking cardiomyocyte renewal. (3) Dysregulation of Cellular Reprogramming: ROS disrupt the transdifferentiation of transplanted cells (e.g., iPSCs) into functional cardiomyocytes, leading to apoptotic cell death and impaired myocardial differentiation, ultimately failing to restore cardiomyocyte populations. Abbreviations: iPSC, induced pluripotent stem cell; mPTP, mitochondrial permeability transition pore; LIPOX, lipoxygenase; MCU, mitochondrial calcium uniporter; OXPHOS, oxidative phosphorylation.

### 2.1. Structural and Functional Mitochondrial Impairment

The heart demands substantial energy to sustain its function, with mitochondrial stability serving as a critical determinant in adult cardiomyocytes—these organelles constitute the core site for energy metabolism and ROS generation [11,19]. Beyond energy production, mitochondria coordinate cellular functions including signaling, calcium homeostasis, oxidative stress modulation, and apoptosis, playing pivotal roles in cell survival and regeneration [19,20]. Under physiological conditions, mitochondria generate low-level ROS primarily via electron leakage from ETC complexes I and III, participating in redox signaling [4,5]. Pathological conditions, however, trigger excessive ROS that damage mitochondrial structure, inducing dysfunction which further amplifies ROS production, establishing a vicious cycle [7,11]. For instance, Silencing mitochondrial energy metabolism genes (e.g., key ETC or TCA cycle genes) via CRISPR/Cas9 or microRNA reduces mitochondrial quantity and ROS levels, markedly promoting cardiomyocyte proliferation [21,22]. This provides direct evidence for the causal relationship between mitochondrial function, ROS imbalance, and regenerative decline.

Excessive ROS drastically diminish mitochondrial energy output, causing cardiomyocyte proliferation blockage and functional impairment. ROS oxidatively inactivate sulfhydryl groups in Fe-S clusters of the respiratory chain and key energy metabolism enzymes, contributing to ETC dysfunction [21]. Concurrently, ROS damage mtDNA, directly impairing the coding of ETC complex I, III, and IV subunits, ultimately resulting in ETC dysfunction and precipitous ATP decline [11,21]. Increased electron leakage from dysfunctional ETC further triggers exponential ROS bursts, forcing cardiomyocytes toward glycolytic energy supply [7,11]. Yet, the limited glycolytic capacity of cardiomyocytes fails to compensate for this energy deficit, leading to systolic failure and proliferative arrest [7,11]. Notably, mtDNA is more vulnerable to ROS than nuclear DNA due to lacking histone protection and limited repair capacity [23]. ROS directly induce 8-oxoguanine modifications and strand breaks in mtDNA, while also oxidatively damaging key base excision repair (BER) enzymes OGG1 and POLG. This reduces mtDNA repair efficiency by over 70%, establishing a positive feedback loop for mutation accumulation [23,24].

ROS inflict structural damage to mitochondria, compromising membranes and cristae while causing kinetic imbalance. This ultimately impaired cell proliferation and cardiac regeneration. Cardiolipin, enriched in mitochondrial membranes, is highly sensitive to ROS-induced lipid peroxidation, which disrupts membrane fluidity and increases permeability. Its end products (e.g., MDA, 4-HNE) covalently modify key ETC proteins (e.g., complex III’s Rieske protein), further impairing ETC function and perpetuating the vicious cycle [25,26]. Additionally, excess ROS synergizes with calcium overload to activate the mitochondrial permeability transition pore (mPTP) [27], inducing matrix swelling, membrane potential (ΔΨm) collapse, proton leakage, reverse ATPase operation, and accelerated energy depletion [20]. Studies demonstrate that inhibiting mPTP opening (e.g., with cyclosporine A) reduces myocardial infarct size [27]. Chronic ROS exposure also disrupts mitochondrial dynamics, inducing fragmentation [28]. Fragmented mitochondria not only lose energy synthesis capacity but also activate the intrinsic immune response via the cGAS-STING pathway, exacerbating the inflammatory microenvironment. This culminates in cardiomyocyte cycle arrest at G1/S phase and complete loss of regenerative potential [29,30].

Collectively, ROS impair mitochondrial structure and function through multidimensional, intertwined mechanisms that constitute a significant barrier to cardiac regeneration. In-depth analysis of the spatiotemporal characteristics and interactions of these mechanisms will provide a theoretical foundation for developing mitochondrion-targeted myocardial protection strategies.

### 2.2. Suppressed Proliferation of Endogenous Cardiomyocytes

The stark contrast in cardiac regenerative capacity across species is largely attributed to evolutionary adaptations in oxygen metabolism and the resultant redox landscape. While hypoxia-tolerant zebrafish maintain robust regeneration into adulthood, the postnatal transition of mammals to an oxygen-rich environment coincides with a significant decline in this reparative potential [18,31]. Puente et al. demonstrated that hypoxic culture conditions enhanced mitotic activity in neonatal mouse cardiomyocytes, evidenced by increased phosphorylation of histone H3 Ser10 (pH3)—a specific G2/M phase marker. Conversely, ROS administration significantly reduced pH3-positive cell populations without inducing apoptosis. Notably, experimental reduction in ROS levels during the early postnatal window effectively delayed cardiomyocyte cell cycle withdrawal, suggesting a regulatory role for ROS in this process [32]. Although this study established a temporal association between DDR activation and cardiomyocyte cell cycle exit, the precise causal role of DDR in physiological cell cycle withdrawal during normal neonatal development remains to be fully elucidated. Notably, this redox-sensitive process is not irreversible: diminishing H_2_O_2_ delays cell cycle exit, whereas hyperoxia accelerates it [18,32].

At the molecular level, ROS precisely modulate cell cycle progression through redox-sensitive signaling nodes. As shown in the neonatal heart model, ROS-induced DNA damage triggers ATM kinase phosphorylation, which regulates G2/M transition by affecting nuclear localization of the CDK1-CyclinB1 complex via redox modification of Wee1 kinase [32,33]. Additionally, ATM phosphorylation activates p53, which upregulates p21 expression. p21 binds CDK2, hindering its interaction with cyclin E and impeding G1/S transition [21]. ROS also function as second messengers activating AKT, which inhibits GSK-3β/CyclinD pathway activation, thereby contributing to G1-phase arrest and suppressing division [34,35]. Oxidative stress signaling interacts with p53-dependent/independent DDR pathway, as well as oxidative effectors Btg1/2 to induce postnatal cell cycle withdrawal. Btg1/2 function as transient repressors within a complex regulatory network, and their deletion (mice with constitutive Btg1/2 deletion or with early neonatal via neonatal AAV9-shRNA knockdown of Btg1/2) promotes transient increases in cardiomyocyte mitotic activity, underscoring their role in the developmental decline of proliferation [36]. Emerging evidence highlights mitochondrial ROS (mROS) as critical regulators of cardiomyocyte proliferation. For instance, mitochondrial dysfunction caused by inactivation of mitochondrial transcription factor A (TFAM) significantly elevates ROS, activates DDR, and induces cardiomyocyte cell proliferative arrest, culminating in fatal cardiomyopathy [22]. Conversely, a more nuanced role for mROS has been revealed: thyroid hormone (T3) stimulates physiological mitochondrial H_2_O_2_ (mH_2_O_2_) production, initiating a noncanonical proliferative cascade: mH_2_O_2_→Prx1→JNK2α2→c-Jun/AP-1→IGF-1→EGF-1→ERK1/2. This pathway effectively bypasses G1/S phase blockade in neonatal mouse cardiomyocytes, expanding our understanding of mROS physiology and identifying novel targets for cardiac regeneration therapies [37].

Emerging evidence confirms that pharmacological strategies, such as the administration of malonate or morroniside, can mitigate these inhibitory signals and reinitiate cardiomyocyte proliferation in adult mammals [38,39]. These findings deepen our mechanistic understanding of ROS signaling in cardiomyocyte proliferation and identify novel intervention targets for cardiac regeneration. Collectively, they establish a multifaceted ROS regulatory network, providing significant theoretical foundations for understanding the equilibrium between cardiomyocyte proliferation and differentiation—a framework that is particularly relevant for developing therapeutic strategies in ischemic heart disease, where pathological ROS elevation drives proliferative failure.

### 2.3. ROS-Mediated Cellular Reprogramming in Non-Cardiomyocytes

Cell-based therapeutic strategies for myocardial regeneration have emerged as promising approaches to restore lost cardiomyocytes. Two principal strategies have been extensively investigated: (1) the transplantation of cardiomyocytes derived from pluripotent stem cells (PSCs), including embryonic stem cells (ESCs) and induced pluripotent stem cells (iPSCs); and (2) direct cellular reprogramming, an artificial process that converts endogenous cardiac fibroblasts—which are abundant in the infarcted heart—directly into induced cardiomyocyte-like cells (iCMs) through the forced expression of cardiac-specific transcription factors or exposure to small molecules [40]. Unlike transplantation, which introduces exogenous cells, direct reprogramming harnesses endogenous cell populations and does not occur naturally in the adult heart; rather, it must be artificially induced.

Both strategies are critically influenced by ROS, which regulate cell survival, fate transition, and differentiation, thereby shaping therapeutic outcomes. Within the hypoxic injury microenvironment, persistently elevated ROS attack transplanted cells, reducing survival and impairing function [41]. Enhancing antioxidant defenses (e.g., superoxide dismutase treatment or N-acetylcysteine pretreatment) increases skeletal myoblast survival post-transplantation by 2-3 fold, underscoring microenvironmental redox homeostasis as pivotal for cell therapy efficacy [42].

ROS modulate epigenetic pathways—including histone/DNA modification, non-coding RNA expression, and ATP-dependent chromatin remodeling—during PSC-to-cardiomyocyte differentiation, thereby influencing cardiovascular disease progression [43,44]. For instance, moderate H_2_O_2_ at the embryoid body stage degrades pluripotency factors Oct4 and Nanog via caspase activation, relieving their HDAC4-mediated transcriptional repression of cardiac genes Gata4 and Nkx2.5 to promote differentiation. Conversely, excessive ROS activates JNK signaling, triggering GATA4 degradation and differentiation blockade [45]. In vitro, elevated ROS cause death in PSC-derived cardiomyocytes (PSC-CMs) [46] and induce protein glutathionylation that disrupts myofibril integrity [47]. Post-transplantation, pathological ROS surges promote non-enzymatic protein degradation or protease activation, damaging the myocardial matrix network [48]. ROS also impair adhesion plaque molecules, hindering transplanted cell attachment—an effect reversed by ROS scavenging [49,50]. Thus, ROS compromise transplant survival through direct oxidative damage and adhesion interference, highlighting microenvironmental redox balance as essential for optimizing stem cell therapy.

Certain antioxidant molecules enhance fibroblast reprogramming to hiPSCs [51], while recent studies reveal ROS significantly regulate direct fibroblast-to-iCM reprogramming. During GMT (Gata4/Mef2c/Tbx5) factor-mediated conversion [52], elevated ROS suppress GMT activity, reducing efficiency to 20–30% of baseline levels. Antioxidants like vitamin C stabilize reprogramming factors and enhance efficiency [53,54], indicating ROS mitigation as critical for successful reprogramming. This regulatory mechanism informs novel strategies to optimize reprogramming systems through precise redox microenvironment control.

Collectively, ROS orchestrate a multidimensional regulatory network in myocardial regeneration: as microenvironmental toxins inhibiting transplanted cell survival; as dose-dependent modulators of fate-determination signaling; and as dynamic balancers of epigenetic remodeling efficiency. Elucidating the spatiotemporal specificity of ROS actions will advance combined antioxidant-cell therapy strategies to achieve breakthroughs in myocardial regeneration efficacy.

### 2.4. Integration of ROS Signaling Networks in Myocardial Regeneration

Collectively, ROS orchestrate a multidimensional regulatory network in myocardial regeneration: as inducers of mitochondrial dysfunction that compromise cellular bioenergetics; as suppressors of endogenous cardiomyocyte proliferation through DNA damage and cell cycle checkpoint activation; and as modulators of cell-based therapeutic strategies, influencing both transplanted cell survival and reprogramming efficiency. These findings deepen our mechanistic understanding of ROS physiology and identify novel intervention targets for cardiac regeneration. Elucidating the spatiotemporal specificity of ROS actions—distinguishing physiological signaling from pathological damage—will be essential for advancing combined antioxidant-cell therapy strategies to achieve breakthroughs in myocardial regeneration efficacy.

## 3. Therapeutic Strategies Targeting ROS for Enhanced Regeneration

**Figure 2:**
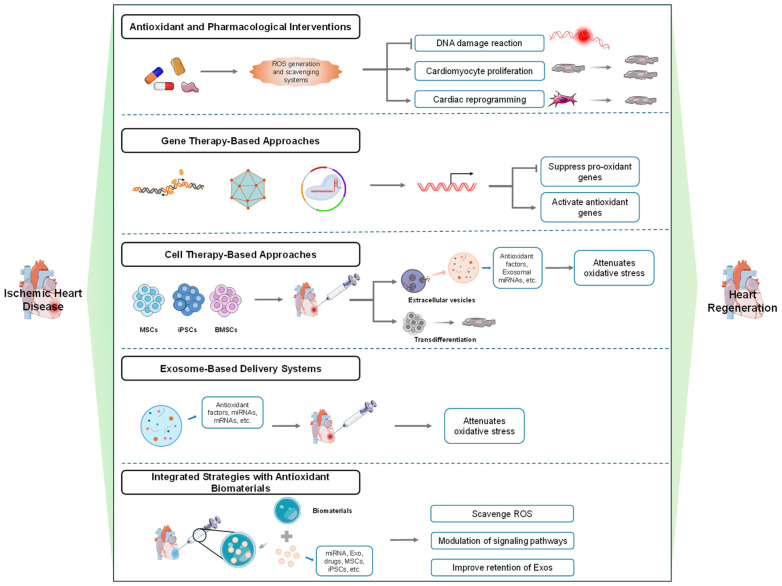
Strategies for ischemic heart disease aiming at heart regeneration, centered on regulating oxidative stress.

The dual role of ROS in myocardial regeneration—as essential signaling molecules under physiological conditions and cytotoxic agents in pathological excess—represents both a challenge and a therapeutic opportunity. While previous sections have outlined the mechanisms of redox-dependent regulation in cardiomyocyte proliferation and mitochondrial homeostasis, translating these insights into effective clinical interventions remains a critical challenge. Current evidence emphasizes that neither global ROS suppression nor unconstrained oxidative stress is beneficial; instead, precise spatiotemporal modulation of specific redox signals is required to promote endogenous repair processes. Emerging approaches seek to selectively target ROS-producing pathways, enhance antioxidant defenses, or deliver redox-modulating agents in a cell-specific manner. The following section explores advanced therapeutic strategies—including pharmacological, genetic, cellular, and biomaterial-based approaches—designed to leverage ROS signaling for functional cardiac regeneration in ischemic heart disease, as illustrated in Figure 2.

This schematic summarizes advanced therapeutic strategies designed to harness ROS signaling for functional cardiac repair. Five integrated modalities are presented: (1) Pharmacological Interventions: Small-molecule agents (represented as capsules and molecular structures) modulate ROS levels via radical scavenging or enzyme targeting, regulating downstream processes such as DNA damage response, cardiomyocyte proliferation, and cellular reprogramming. (2) Gene Therapy: Nucleic acid-based tools (e.g., oligonucleotides, viral vectors) mediate transcriptional regulation to inhibit pro-oxidant genes or enhance antioxidant defenses, correcting oxidative stress and establishing a pro-regenerative microenvironment. (3) Cell Therapy: Transplanted cells—including MSCs, iPSCs, and BMSCs—hone to injury sites and facilitate repair through paracrine secretion of antioxidants (via extracellular vesicles/exosomes) and direct differentiation into cardiac lineages. (4) Exosome-Based Delivery: Engineered exosomes loaded with bioactive molecules (e.g., miRNAs, mRNAs, drugs) serve as targeted vehicles to mitigate local oxidative stress and enhance cardiomyocyte survival and proliferation. Antioxidant Biomaterials: Functionalized biomaterials (e.g., microgels, protein scaffolds) are integrated with therapeutic agents (drugs, cells, exosomes) to synergistically scavenge ROS, modulate signaling pathways, and improve cell retention, thereby promoting regenerative outcomes.

### 3.1. Antioxidant and Pharmacological Interventions

Excessive ROS accumulation induces oxidative stress and DNA damage, key factors inhibiting cardiomyocyte proliferation. Antioxidant molecules demonstrate positive effects on both proliferation and cardiac regeneration. Exogenous antioxidants reduce oxidative DNA damage, inhibit DDR-mediated cell proliferative arrest, and significantly promote cardiomyocyte proliferation and regeneration [32,55]. Current antioxidant therapies targeting ROS regulation comprise three classes: natural compounds, synthetic molecules, and enzymatic systems. For example, malonate inhibits succinate dehydrogenase (SDH), suppressing mitochondrial ROS during ischemia to promote metabolic reprogramming and regeneration [39]. The small molecule TT-10 exemplifies this dual functionality, activating NRF2 signaling to reduce ROS levels, inhibit DNA damage, and promote myocardial regeneration [56]. Similarly, carpaine—a papaya leaf alkaloid—stimulates H9c2 cell proliferation via mitochondrial membrane potential stabilization, ROS reduction, and cell cycle protein upregulation (cyclin D1, PCNA) through FAK-ERK1/2 and FAK-AKT pathway activation [57]. Multitarget flavonoids like fisetin demonstrate therapeutic promise by attenuating ROS production, inhibiting caspase activation, and upregulating pro-proliferative genes in myocardial ischemia–reperfusion models [58]. Sodium hydrosulfide (NaHS) further illustrates this paradigm, enhancing endogenous defenses via SOD2 upregulation to reactivate cardiomyocyte cycling and improve post-infarct regeneration in neonatal mice [59].

Antioxidants also critically enhance cardiac reprogramming efficiency—a persistent bottleneck in regeneration strategies [52,60]. Research has demonstrated that antioxidants can promote the direct reprogramming of fibroblasts into cardiomyocytes and enhance the efficiency of this process [61]. Selenium supplementation synergizes with microRNA cocktails (miR-1, miR-133, miR-208, miR-499), boosting fibroblast-to-cardiomyocyte transdifferentiation efficiency while upregulating cardiac markers (cTnI, Cacna1c) through Nanog-mediated antioxidant pathways [62,63]. Vitamin C demonstrates analogous benefits, amplifying GMT (Gata4/Mef2c/Tbx5) reprogramming efficiency and cardiac gene expression (TNNT2, ACTC1) in 2D/3D models via antioxidant-dependent epigenetic remodeling (histone demethylation/acetylation) [64]. Crucially, non-antioxidant structural analogs lack this activity, confirming redox regulation as the primary mechanism [53].

Emerging biomaterial strategies complement these approaches. Decellularized myocardial matrix hydrogels simultaneously mitigate oxidative damage and foster DNA synthesis/cell cycle activation microenvironments, offering novel regenerative therapeutic platforms [65]. Collectively, integrating ROS-modulating antioxidants with biomaterial engineering represents a transformative strategy to enhance myocardial regeneration and functional recovery.

### 3.2. Gene Therapy-Based Approaches

Gene therapy offers novel strategies to improve the cardiac microenvironment through direct intervention in ROS generation and scavenging systems via targeted gene or non-coding RNA delivery. Current approaches primarily focus on activating antioxidant genes or suppressing pro-oxidant genes to modulate ROS levels for cardiac regeneration. Non-coding RNAs—particularly microRNAs (miRNAs)—have garnered significant attention for their regulatory roles in cardiovascular development and disease, with notable advances in understanding miRNA-mediated oxidative stress responses post-cardiac injury [66,67]. Studies identify specific non-coding RNAs (miR-323-3p, miR-340-5p, miR-222-3p, miR-1825, MHRT, circSamd4) that critically influence cardiomyocyte redox homeostasis [68,69,70]. For example, miR-1825 promotes cardiomyocyte proliferation by suppressing ROS generation and attenuating oxidative stress/DNA damage, while also inducing ischemic myocardial regeneration, reducing infarct size, and improving cardiac function in LAD ligation model [68]. The mitochondria-localized circSamd4 reduces oxidative stress and DNA damage by facilitating Vcp protein mitochondrial translocation and downregulating Vdac1 expression to stabilize mitochondrial permeability transition pores, ultimately promoting myocardial proliferation, reducing apoptosis, and enhancing cardiac function [70].

Beyond non-coding RNA delivery, gene editing enables precise long-term ROS modulation. p16INK4a overexpression triggers aberrant ROS accumulation that activates autophagy and inhibits CDK4/6/CyclinD1 pathway activity, suppressing myocardial regeneration; antioxidant N-acetylcysteine rescues CDK4/6/CyclinD1 signaling and restores proliferative capacity [71]. Similarly, Nrf3 knockdown or Pitx2 overexpression reduces mitochondrial ROS, improving cardiomyocyte survival and proliferation [72]. AAV9-mediated Btg1/2 knockdown during embryonic/neonatal stages transiently extends the cardiomyocyte proliferation window to postnatal days 7–14. As downstream effectors of p53-dependent and independent DDR pathways, Btg1/2 may regulate postnatal cardiomyocyte cell cycle exit by modulating oxidative stress (e.g., prolonging regeneration under hypoxia) and cellular homeostasis [36]. Breakthrough studies reveal that Tert gene activation reverses ROS-induced cardiomyocyte proliferative arrest and promotes neovascularization via dual mechanisms: hnRNPA2B1–Tert–telomere protection axis and NF-κB/VEGF signaling. Targeted Tert or hnRNPA2B1 overexpression effectively promotes post-MI regeneration, establishing a novel repair pathway [73].

Collectively, gene therapies leveraging non-coding RNA delivery, antioxidant gene regulation, and oxidative stress-targeted interventions demonstrate substantial potential for mitigating ischemia–reperfusion injury and promoting cardiac regeneration/functional recovery. These innovative approaches deepen mechanistic insights into ROS-related cardiac regeneration while expanding translational options. Future work should optimize delivery systems, enhance targeting specificity, and explore combinatorial interventions to advance gene therapy applications in cardiovascular regenerative medicine.

### 3.3. Cell Therapy-Based Approaches

Cell therapy represents a strategic approach to enhance cardiac regeneration, often by indirectly modulating the oxidative microenvironment through paracrine signaling. Among various cell sources, mesenchymal stem cells (MSCs) have emerged as pivotal players due to their multipotent differentiation capacity and robust paracrine activity, which includes the secretion of antioxidant factors and exosomal miRNAs [74].

Despite encouraging preclinical results, the clinical translation of MSC therapy for cardiac repair has yielded largely disappointing outcomes [75]. A recent meta-analysis of clinical trials in patients with acute myocardial infarction demonstrated that while MSC treatment significantly improved left ventricular ejection fraction (LVEF) within the first 12 months post-infarction, no significant effect on LVEF was observed beyond 12 months, and MSC therapy did not significantly reduce the incidence of major adverse cardiac events (MACE) [76]. Another comprehensive 20-year review of MSC therapy for ischemic cardiomyopathy found that although some functional improvements were noted, 33.3% of studies showed no significant difference between MSC-treated and control groups, and the overall quality of evidence was limited by small sample sizes and study heterogeneity [77]. These findings underscore the gap between preclinical promise and clinical efficacy, highlighting the need for optimization strategies.

Mechanistic studies have demonstrated that MSC administration through intravenous, coronary, or intramyocardial routes in myocardial infarction models can mitigate inflammatory cascades, reduce fibrotic remodeling, and enhance ventricular performance [75]. Strategic optimization of delivery methods, particularly through pre-transplantation systemic intravenous injection, has been shown to improve myocardial retention of exogenous cells and amplify therapeutic efficacy [74]. Mechanistic studies in D-galactose-induced senescent rat models reveal that MSC transplantation attenuates oxidative stress through ROS/inflammasome axis modulation, potentially mediated by exosomal miRNA-mediated inhibition of gp91phox (NOX2 subunit), ultimately improving cardiac functional recovery and tissue regeneration [78,79].

Despite their therapeutic benefits, MSC survival and differentiation efficiency are significantly compromised in high-ROS microenvironments [80]. To overcome these limitations, innovative strategies involving antioxidant preconditioning and genetic modification have been developed. Pharmacological pretreatment with flavonoid antioxidants (e.g., rutin and quercetin) enhances MSC resilience by activating PI3K/Akt signaling pathways while suppressing H_2_O_2_-mediated ROS generation, thereby improving both cell survival and cardiac functional recovery [81]. Genetic engineering approaches, particularly microRNA modulation, have shown remarkable efficacy in MSC optimization. Let-7b transfection significantly attenuates ROS-induced apoptosis via caspase-3 pathway inhibition, substantially improving post-transplantation outcomes as demonstrated in multiple experimental models [82]. Parallel advancements include the synergistic application of bone marrow-derived MSCs with edaravone, a free radical scavenger, which enhances cellular hypoxia resistance while potentiating angiogenic and cardiomyogenic regeneration through Akt pathway activation—a promising therapeutic paradigm for acute myocardial infarction management [83].

In summary, cell-based interventions hold substantial potential for cardiac regeneration through direct and indirect ROS regulation. Strategic optimization via antioxidant priming or miRNA modification enhances cell survival and therapeutic precision, advancing clinical translation prospects. Future research should prioritize elucidating optimal delivery modalities, paracrine mechanisms, and host-microenvironment interactions to accelerate regenerative cardiology applications.

### 3.4. Exosome-Based Delivery Systems

Exosomes have emerged as pivotal mediators of intercellular communication, with secretion observed across various cell types including mesenchymal stem cells and endothelial cells. Their bilayered membrane architecture effectively shields bioactive cargo (proteins, nucleic acids [miRNAs, mRNAs], and lipids) from degradation while enabling targeted biomolecule delivery [79]. This intrinsic stability and targeting specificity position exosomes as optimal therapeutic vectors for precise ROS modulation and cardiac regeneration [84,85]. Experimental evidence demonstrates that hypoxia-preconditioned cardiovascular microvascular endothelial cell exosomes (CMECs H-exo) significantly enhance hypoxia/reoxygenation-treated H9C2 cell proliferation. Mechanistic investigations revealed that exosomal miR-210-3p orchestrates cardioprotection by directly targeting transferrin receptor (TFR), effectively suppressing iron-mediated cell death pathways, attenuating lipid ROS accumulation, and ultimately promoting cardiomyocyte survival and proliferation [86].

In addition to leveraging the bioactive components inherent in natural exosomes, the engineering of modified exosomes offers novel approaches for cardiac regenerative therapy. The researchers developed an innovative exosome delivery system: The Mn3O4 nanoparticles were preloaded into mesenchymal stem cell-derived vesicles (Mn@EV), which were endowed with antioxidant activity similar to peroxisomes. The further immobilization of lactate oxidase (LOX) on the vesicle surface has been shown to catalyze the breakdown of lactate that has accumulated under conditions of stress. This dual-function design effectively maintains myocardial redox homeostasis by synchronizing the scavenging of ROS and lactate, inhibiting the DDR pathway, and promoting myocardial regeneration while protecting cardiac function [87]. While MSC-derived extracellular vesicles (MSC-EVs) demonstrate cardioprotective, immunomodulatory, and pro-regenerative properties [61,88], their clinical translation is hindered by suboptimal targeting and delivery efficiency [89]. The aforementioned engineered systems (Mn@EV, NA@MEV) exemplify progress in overcoming these barriers through modular design integrating targeting motifs, stimulus-responsive release, and multimodal therapeutic cargo.

Exosome therapeutics hold transformative potential for cardiac regeneration via natural cargo delivery or engineered multifunctionality. Prioritizing research on tissue-specific targeting, host-microenvironment interactions, and scalable production will accelerate clinical translation. Synergistic integration with gene editing or biomaterial platforms may further enhance spatiotemporal control over ROS modulation and regenerative outcomes.

### 3.5. Integrated Strategies with Antioxidant Biomaterials

ROS-responsive biomaterials have emerged as promising tools for cardiac regeneration, leveraging unique physicochemical properties to dynamically respond to local microenvironmental ROS fluctuations [90]. These materials promote tissue repair through direct ROS scavenging or modulation of associated signaling pathways [91]. Advanced material systems exemplify these therapeutic strategies. An injectable polydopamine-ginsenoside Rb1 hydrogel demonstrates microenvironment-responsive uncrosslinking in myocardial acidic/oxidative conditions, simultaneously scavenging ROS, preserving mitochondrial integrity, and suppressing STING pathway-mediated M1 macrophage polarization to enhance cardiac function and promote regeneration [92]. Another innovative system employs diselenide bond-containing hydrogels (bond energy 172 kJ/mol) that combine potent ROS clearance with miR-19a/b delivery, synergistically activating PI3K/Akt/Wnt pathways while suppressing CYBA expression-enabling dual regulation of cardiomyocyte cycle re-entry through oxidative microenvironment modulation [93].

The multifunctional carrier potential of these materials extends to stem cell-based therapies. A graphene oxide/alginate composite significantly enhances human MSC survival and paracrine effects under oxidative stress, promoting cardiomyocyte maturation and regeneration-associated cytokine expression through superior antioxidant capacity [94]. Similarly, oxidized hyaluronic acid-polylysine (OHA-PL) hydrogels loaded with adipose-derived MSC exosomes (ADSC-Exos) that sequentially: scavenge early post-MI ROS, modulate macrophage polarization to reduce inflammation, inhibit late-stage fibrosis/ventricular remodeling, and promote angiogenesis/electrophysiological recovery. These collective capabilities establish ROS-responsive biomaterials as promising therapeutic platforms for cardiac tissue engineering and regenerative medicine [95]. Recent innovations integrate bioactive peptides with responsive delivery systems. A methacrylated gelatin hydrogel co-encapsulating marine-derived antioxidant peptide MMP12 and pro-angiogenic peptide KRX achieves sustained retention in the infarcted area through minimally invasive implantation. This dual-action system synergistically combines oxidative stress mitigation with neovascularization promotion, demonstrating enhanced myocardial repair in preclinical models [96].

These advancements highlight the unique capacity of ROS-responsive biomaterials to integrate antioxidant, immunomodulatory, and regenerative functions through combinatorial therapeutic approaches. Future development should prioritize optimization of three key parameters: (1) Dynamic responsiveness to redox gradients, (2) Biocompatibility for long-term implantation, and (3) Spatiotemporal control of therapeutic agent release essential milestones for clinical translation in post-infarction cardiac repair.

## 4. Discussion and Conclusions 

ROS act as central orchestrators in cardiovascular pathophysiology, exhibiting a dual role as signaling molecules and cytotoxic agents that presents a therapeutic paradox. The high metabolic activity and relatively limited antioxidant defenses in cardiovascular tissues create a microenvironment highly susceptible to ROS-mediated damage. In myocardial regeneration, ROS-induced genomic instability is recognized as a critical barrier to effective therapy. Experimental models reveal that oxidative DNA lesions—such as 8-oxoguanine accumulation—elevate genomic instability indices by 3- to 5-fold, impairing the proliferation of cardiac precursor cells. For instance, studies using H9c2 cells—an immortalized cell line derived from embryonic rat heart tissue that retains certain cardiomyocyte-like properties—have demonstrated that oxidative DNA damage compromises cell viability and proliferative potential [97]. Although antioxidant strategies show regenerative benefits in preclinical studies, their clinical application faces a precision challenge: conventional antioxidants disrupt essential ROS-mediated signaling. Physiological H_2_O_2_ gradients (10–100 nM) activate AKT-dependent angiogenesis, while controlled superoxide levels regulate NF-κB-mediated immune surveillance—both processes are compromised by non-selective scavenging.

Mitochondria-targeted antioxidants (e.g., MitoQ, SkQ1) represent a paradigm-shifting strategy by neutralizing mitochondrial ROS while preserving cytoplasmic redox signaling [98,99]. Their preclinical success in improving post-infarct functional recovery and promoting coronary neovascularization underscores significant therapeutic potential. However, clinical translation requires addressing three key challenges: (1) achieving precise modulation of mitochondrial subcompartment ROS gradients without disrupting oxidative phosphorylation; (2) developing cardiovascular-specific delivery systems to minimize off-target effects; and (3) establishing personalized dosing regimens that balance regenerative efficacy with long-term biosafety.

## Data Availability

No new data were created or analyzed in this study.
